# Preventing Substance Use Among Indigenous Adolescents in the USA, Canada, Australia and New Zealand: a Systematic Review of the Literature

**DOI:** 10.1007/s11121-019-01038-w

**Published:** 2019-10-22

**Authors:** Mieke Snijder, Lexine Stapinski, Briana Lees, James Ward, Patricia Conrod, Christopher Mushquash, Lorenda Belone, Katrina Champion, Cath Chapman, Maree Teesson, Nicola Newton

**Affiliations:** 1 The Matilda Centre, Faculty of Health and Medicine, The University of Sydney, Darlington, Sydney, NSW 2006 Australia; 2 South Australian Health and Medical Research Institute, Flinders University, Adelaide, Australia; 3 Department of Psychiatry, Université de Montréal, Montreal, Canada; 4 Centre for Rural and Northern Health Research, Northern Ontario School of Medicine, Lakehead University, Thunder Bay, Canada; 5 Department of Health, Exercise & Sports Sciences, College of Education, University of New Mexico, Albuquerque, NM USA

**Keywords:** Substance use, Prevention, Indigenous, Native, Aboriginal, Adolescent, Evaluation

## Abstract

This systematic review assessed the current evidence base of substance use prevention programs for Indigenous adolescents in the USA, Canada, Australia and New Zealand. The authors investigated (a) the outcomes, type, setting and context of prevention programs; (b) the common components of beneficial prevention programs; and (c) the methodological quality of evaluations of included prevention programs. The authors searched eight peer-reviewed and 20 grey literature databases for studies published between 1 January 1990 and 31 August 2017. Data extracted included type of program (culturally adapted, culture-based or unadapted), the setting (school, community, family or multi-setting), delivery (computerised or traditional), context (Indigenous-specific or multi-cultural environment) and common components of the programs. Program evaluation methodologies were critically appraised against standardised criteria. This review identified 26 eligible studies. Substance use prevention programs for Indigenous youth led to reductions in substance use frequency and intention to use; improvements in substance-related knowledge, attitudes and resistance strategies; and delay in substance use initiation. Key elements of beneficial programs included substance use education, skills development, cultural knowledge enhancement and community involvement in program development. Five programs were rated as methodologically strong, seven were moderate and fourteen were weak. Prevention programs have the potential to reduce substance use among Indigenous adolescents, especially when they are developed in partnership with Indigenous people. However, more rigorously conducted evaluation trials are required to strengthen the evidence base.

## Introduction

Indigenous peoples in the United States of America (USA), Canada, Australia and New Zealand have a comparable history of colonisation and dispossession of culture and land by English settlers, resulting in predominantly English-speaking countries in which Indigenous People are a marginalised minority. The centuries following early colonisation have continued to harm Indigenous peoples through *cultural genocide* and forced assimilation attempts (e.g. residential schools and the 60s Scoop in Canada, and child removal policies leading to Stolen Generations in Australia). Some governments have officially acknowledged and apologised for their roles in the disruption and abuses inflicted upon Indigenous peoples (Coalition of Australian Governments 2009; Truth and Reconcilitation Commission 2015).

This history combined with contemporary issues such as continued policy failures in social services, education and health care systems have resulted in loss of cultural knowledge and language in Indigenous communities and poorer outcomes in many of the social determinants of health compared with the non-Indigenous population, and significant trauma for Indigenous peoples with lasting inter-generational effects (Cornell 2006; King et al. 2009). The impact of these previous traumatic events on family structures and high level of substance use reported among Indigenous adults has left its mark on Indigenous adolescents, who, consequently, experience higher levels of psychological distress as well as an increased susceptibility to substance use and related harms, compared to their non-Indigenous counterparts. For example, binge drinking (consuming 5 or more drinks in one session) rates among Indigenous adolescents in the USA were up to five times higher than all other ethnicities (Centers for Disease Control and Prevention 2018). While rates of tobacco smoking are reducing, Indigenous adolescents, in the USA are nine times more likely to smoke (Centers for Disease Control and Prevention 2018), those in New Zealand three times more likely (Ministry of Health 2015) and those in Canada twice as likely (Reading and Wien 2009) compared to non-Indigenous adolescents. Indigenous adolescents are also more likely to report cannabis use: five times higher in Canada and in the USA (Beauvais 1992). Injecting drug rates are three times higher among Indigenous adolescents compared to non-Indigenous adolescents in Australia (Bryant et al. 2016) and the USA (Centers for Disease Control and Prevention 2018). Furthermore, Indigenous adolescents are likely to commence drug use 2 to 6 years younger compared to their non-Indigenous counterparts (Australian Institute of Health and Welfare 2006). Early onset, and escalation, of substance use among Indigenous adolescents have been identified as risk factors for substance-related disorders and associated problems such as poorer educational outcomes and comorbid mental health problems, later in life (Behrendt et al. 2009; Degenhardt et al. 2016; Kunitz 2008; Whitesell et al. 2009; Windle et al. 2008). Prevention of adolescent substance use has therefore been identified as a key strategy to improve Indigenous wellbeing (Australian Government 2013; Dickerson et al. 2018; King et al. 2009).

Substance use prevention strategies have shown to be effective for non-Indigenous adolescents, including school-based, community-based and family-based programs (Foxcroft and Tsertsvadze 2012; Newton et al. 2017). Given the unique historical and cultural contexts, non-Indigenous programs likely require a cross cultural translation for Indigenous adolescents, mapped against different communication styles and language, accounting for situational and place context, and different perspectives of health and identity (Castro and Yasui 2017; Dickerson et al. 2018). Programs adapted from existing non-Indigenous programs (culturally adapted programs) or developed specifically for the local Indigenous cultural context (culture-based programs) are likely to be effective in the prevention of alcohol and other drug use (Belone et al. 2017; Dickerson et al. 2018; Leske et al. 2016).

To date, a comprehensive synthesis of the international evidence for Indigenous substance use prevention programs has not been conducted. This systematic review will address this gap by reviewing the effectiveness of substance use prevention programs for Indigenous adolescents in the USA, Canada, Australia and New Zealand. More specifically, for Indigenous adolescents, this review will investigate (a) the outcomes, type (culturally adapted, culture-based, unadapted), setting (community, school, family) and context (multi-cultural, Indigenous-specific) of prevention programs; (b) the common components of beneficial substance use prevention programs; and (c) the methodological quality of evaluations of substance use prevention programs.

## Methods

### Search Strategy

**Fig. 1:**
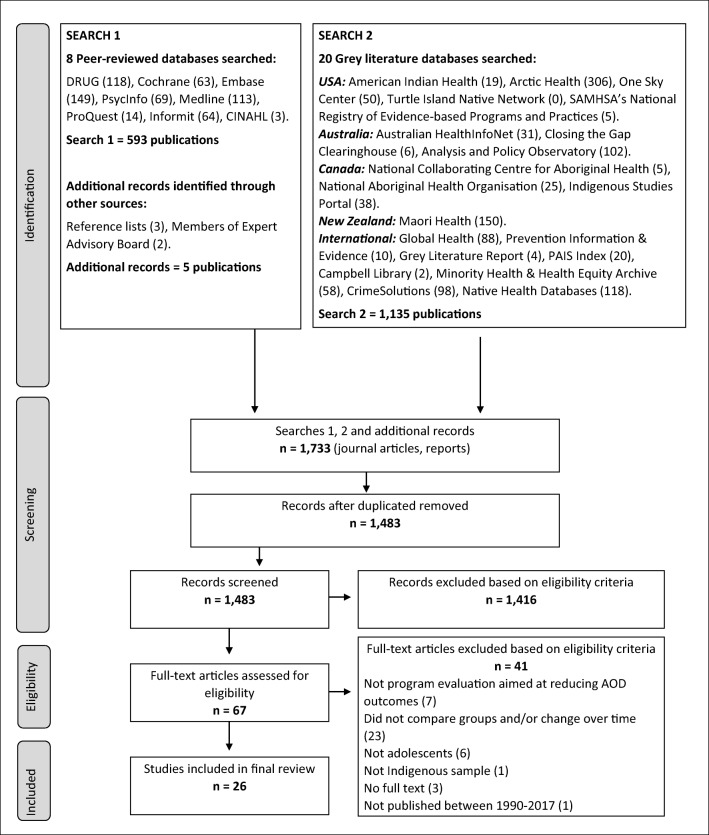
PRISMA flow diagram: systematic search strategy to identify studies evaluating substance use prevention programs for Indigenous youth

This systematic review followed the Preferred Reporting Items for Systematic Review and Meta-Analysis (PRISMA) guidelines (Moher et al. 2009) and a pre-specified, published protocol (Snijder et al. 2018; PROSPERO registration number: CRD42017081885). Figure 1 summarises the complete study selection process. A detailed description of the methods can be found in Snijder et al. (2018). Twenty-eight electronic databases were searched using search terms developed to identify evaluations of substance use prevention programs for Indigenous adolescents in the USA, Canada, Australia and New Zealand (Appendix Table 3). Reference lists were manually searched, and publications were received from researchers in the field.

Studies were included if they (a) evaluated a prevention program aiming to reduce substance use and related outcomes, (b) compared an experimental group to a control group and/or assessed changes over time, (c) included participants aged 10 to 19 years, (d) comprised at least 50% of participants who identified as Indigenous and/or there was a specific sub-analysis for Indigenous participants, (e) were published between 1 January 1990 and 31 August 2017, and (f) full text was available to the authors.

BL screened all titles and abstracts based on the eligibility criteria, and a random selection of 25% of studies was independently screened by MS to ensure accuracy in the study selection. There was an agreement on 90% of studies, and consultation was held between the two authors to reconcile the disagreements. MS and BL independently assessed the eligibility of 67 full-text articles, with high inter-rater agreement between the two authors for this assessment (96%; κ = 0.829). Again, consultation was held between the two authors to reconcile differences of opinion.

### Data Extraction and Analysis

Data relating to the program and the evaluation of the program were extracted from the included studies. Program types were culture-based, culturally adapted or unadapted (Leske et al. 2016). Program setting comprised school, community, family-based programs or multi-setting programs (Lee et al. 2013). Program context was defined as being conducted an Indigenous-specific environment (e.g. reservation) or a multi-cultural environment (e.g. public-school classroom).

Data relating to the evaluation of the program included the sample size and composition, the study design and substance-related and non-substance-related outcomes. Substance-related outcomes were substance use frequency, substance-related knowledge, attitudes towards substances, substance resistance strategies, intention to use and substance use initiation (Lee et al. 2013). Due to the heterogeneity of study designs and outcomes, a narrative synthesis of the results is provided, rather than a meta-analysis.

Programs were identified as beneficial if there were beneficial effects on more than 50% of substance-related outcomes measured in the study. “Beneficial effects” are defined as any positive changes reported in the study (e.g. statistically significant improvements, percentage changes, qualitatively recorded improvements). Common program components were determined by identifying the components of these programs that showed broader beneficial impacts.

### Critical Appraisal of Evaluation Methodology

The methodological quality of quantitative studies was assessed using the Quality Assessment Tool for Quantitative Studies from the Effective Public Health Practice Project (EPHPP) (Thomas et al. 2004), and the methodological quality of qualitative study components was assessed using a modified version of the qualitative tool by Long and Godfrey (2004). Assessment of both quantitative and qualitative studies was conducted by BL. A random selection of 25% of studies was independently appraised by MS; there was 100% agreement.

## Results

Of the 1483 articles screened, 26 publications reporting results of evaluations of substance use prevention programs for Indigenous youth were eligible. Reasons for exclusion are detailed in Fig. 1. Of the 26 publications, 24 studies evaluating 27 prevention programs were identified from peer-reviewed databases and two studies were identified from the grey literature. Eighteen (70%) studies were conducted in the USA, six (23%) in Australia and two (8%) in Canada, while no studies in New Zealand met the eligibility criteria. Thirteen programs targeted multiple substances: alcohol was the most commonly targeted substance (*n* = 15), followed by tobacco (*n* = 12) and cannabis (*n* = 10). Other substances included stimulants (*n* = 1), inhalants (*n* = 2) and analgesics (*n* = 1). Appendix Table 3 provides an overview of all included studies, the evaluated programs and the evaluation outcomes.

### Outcomes, Type, Setting and Context of Prevention Programs for Indigenous Adolescents

Outcomes of the evaluations are listed by substance-related outcome type in Table 1. Frequency of use was measured in 73% of studies, 39% measured substance-related knowledge, 19% measured attitudes towards substances, and 8% measured substance resistance strategies, intention to use and substance use initiation. Beneficial outcomes were found for 50 to 100% of studies across all outcome types. In total, 14 programs were found to be beneficial with positive effects on more than 50% of measured substance-related outcomes.

**Table 1:** Outcomes measured in included studies

	Iatrogenic	Null	Beneficial
Substance use frequency (*n* = 19)	1 (6%)	8 (38%)	10 (56%)
Substance-related knowledge (*n* = 10)	0	4 (20%)	7 (60%)
Attitudes towards substances (*n* = 5)	0	2 (33%)	3 (67%)
Substance resistance strategies (*n* = 2)	0	1 (50%)	1 (50%)
Intention to use (*n* = 2)	0	0	2 (100%)
Substance use initiation (*n* = 2)	0	1 (50%)	1 (50%)

In terms of program type, fifteen (58%) studies evaluated a culture-based program, ten (38%) studies evaluated a culturally adapted program and one (4%) study evaluated an unadapted program. Cultural adaptation of non-Indigenous programs included translation of concepts into local Indigenous language and concepts, developing cultural images and the adaptation of activities to include cultural activities such as prayer, dancing and circle conversations. Three studies evaluated the *Keepin’ it REAL* program: two were a cultural adaptation of this program called *Living in 2 worlds* (Kulis et al. 2013; Kulis et al. 2016), and one was unadapted (Dixon et al. 2007). Dixon et al. (2007) found iatrogenic outcomes for Indigenous adolescents, namely an increase in cannabis use following the program. The pilot of the adapted program produced beneficial outcomes for substance resistance strategies (Kulis et al. 2013). The efficacy trial showed improvements in substance-related knowledge, but no statistically significant improvements in substance resistance strategies or substance use frequency (Kulis et al. 2016).

In terms of program setting, thirteen (50%) evaluated programs were school-based, five (19%) were community-based, five (19%) combined school and family, one (4%) combined community and school, two (8%) combined family and community-based programs, and four (15%) were delivered in community, family and school settings. Family involvement in programs was primarily through one-off workshops or pamphlets given to parents and was not specifically evaluated in any study. Two studies evaluated programs that were delivered in a single setting compared to multiple settings: Schinke et al. (2000) compared a school-based program to the same program combined with a community-based program and Komro et al. (2017) compared a school-based program (*CONNECT*), a community-based program (*CMCA*) and a program where *CONNECT* and *CMCA* were combined. These studies found the multi-setting programs had a smaller effect on substance use than the school- or community-based program on their own (Komro et al. 2017), or that there was no added benefit of a community component to the school-based program (Schinke et al. 2000).

In terms of the context in which prevention programs were implemented, 12 (46%) were implemented in reservation/discrete Indigenous communities where all community members identify as Indigenous, ten (38%) were implemented in an urban setting, two in Indian territory and two in rural communities. Programs implemented in non-Indigenous specific areas still had 100% Indigenous participants in the evaluation study, except for five studies which had between 16 and 90% Indigenous participants and were all school-based (Carter et al. 2007; Dixon et al. 2007; Komro et al. 2017; Malseed et al. 2014; Petoskey et al. 1998).

### Common Components in Effective Substance Use Prevention Programs for Indigenous Youth

Table 2 lists which components of prevention programs had beneficial effects on which substance-related outcome for Indigenous youth. Nine (64%) beneficial programs were developed by, or together with, the community. Community involvement included parents, youth, community leaders, parents and other community members providing input in program development and feedback on versions of the program. Nine (64%) beneficial programs incorporated cultural knowledge enhancement, including integration of cultural activities (e.g. ceremonies, storytelling, rituals, dancing), learning about traditional beliefs and practices, integration of culturally specific concepts and use of culturally appropriate artwork and designs. Eleven (79%) beneficial programs had a skill development component, which included problem-solving, substance resistance strategies, interpersonal skills, decision-making and self-management skills. Substance use education components were included in eight (57%) beneficial programs and included information on the effects of substances, short- and long-term consequences and information about addiction.

**Table 2:** Components of prevention programs leading to beneficial substance-related outcomes amongst Indigenous youth

	Substance use frequency (*n* = 10)	Substance-related knowledge (*n* = 7)	Attitudes towards substances (*n* = 3)	Substance resistance strategies (*n* = 1)	Intention to use (*n* = 2)	Substance use initiation (*n* = 1)
	*N* (%)	*N* (%)	*N* (%)	*N* (%)	*N* (%)	*N* (%)
Community resource development Elders, parents, students, community leaders and members	5 (50%)	5 (71%)	3 (100%)	1 (100%)	2 (100%)	1 (100%)
Cultural knowledge enhancement Traditional values, concepts, ceremony, storytelling, ancestry, prayer	9 (90%)	4 (57%)	2 (66%)	1 (100%)	2 (100%)	1 (100%)
Skill development Goal setting, problem-solving, decision-making, peer support, communication, assertiveness, resilience, interpersonal, occupational, AOD resistance skills	10 (100%)	4 (57%)	2 (66%)	1 (100%)	–	1 (100%)
Indigenous facilitators Local Indigenous community members received training	4 (40%)	3 (42%)	1 (33%)	1 (100%)	–	1 (100%)
Substance use education Effects of use, addiction	6 (60%)	4 (57%)	2 (66%)	–	1 (50%)	–
Trained worker/teacher facilitation Social workers, teachers or youth workers received training in specific program	5 (50%)	3 (42%)	–	–	1 (50%)	–
Health education Holistic concepts of health, physical activity, nutrition	3 (30%)	2 (29%)	–	–	–	1 (100%)
Mental health education Self-talk, depression, suicide, identifying personal strengths, stress management	2 (20%)	–	–	–	–	–
Relationships Importance of community, family, role models, family conflict management	2 (20%)	–	–	–	–	–
Recreational Sport, festivals, painting, discos, film-making	2 (20%)	2 (29%)	–	–	–	–
Booster session Repeating key messages 3 to 6 months later	2 (20%)	–	1 (33%)	–	–	–
Computerised delivery Online-based program	–	–	1 (33%)	–	1 (50%)	–

In addition to these four most commonly used components, other components included the use of facilitators trained in program delivery (6 programs) and Indigenous program facilitators (5 programs). Four programs used health education strategies, which included providing healthy alternatives to substance use, incorporating a holistic concept of wellbeing and media campaigns about healthy lifestyles. Three programs included a booster session 3 to 6 months following program implementation and three programs included recreational activities, such as sports, festivals, painting, going out bush and filmmaking.

Two programs were delivered online: *SmokingZine* and *Boy and Woman Bear. SmokingZine* was an adaptation of a non-Indigenous Web site and included educational modules with culturally relevant content and imagery. It was found to reduce intention to use tobacco, reduce positive beliefs about tobacco smoking, and increase likelihood to help others quit smoking (Bowen et al. 2012). *Boy and Woman Bear* was an illustrated story presented on a computer; the evaluation found no improvements in tobacco knowledge (Schinke et al. 1994).

### Methodological Quality of Substance Use Prevention Program Evaluations Among Indigenous Youth

All 26 included studies used quantitative evaluation methods and four studies also included a qualitative component (Baydala et al. 2014; Donovan et al. 2015; Gray et al. 1998; Lee et al. 2008).

#### Quantitative Study Components

Appendix Table 4 shows the outcome of the methodological quality assessment of quantitative studies. Five (19%) studies had a strong quality rating according to the quality assessment tool from EPHPP, seven (27%) had a moderate rating and fourteen (54%) studies had a weak rating. Participants were deemed likely to be representative of the population in three (12%) studies. Five (19%) studies were RCTs, three (12%) studies were controlled clinical trials, five (19%) studies were cohort clinical trials and thirteen (50%) studies were cohort studies. Confounding factors were discussed in all studies; 16 studies did not find significant confounding factors and seven (27%) studies controlled for significant confounding variables. Thirteen (50%) studies used validated outcome measurement tools and thirteen (50%) studies used reliable measurement tools. Withdrawals and drop-outs were reported in fourteen (54%) studies. Program completion rates were recorded in fourteen (54%) studies. Program fidelity was measured in nine (35%) studies (Table 5).

#### Qualitative Study Components

All four studies with a qualitative component provided some description of the data collection and analysis methods. Detailed data collection and analysis descriptions (e.g. participant recruitment, focus group procedures and a clear description of the data that was recorded) were provided in two of the four (50%) studies (Baydala et al. 2014; Lee et al. 2008). The potential for researcher bias was discussed in one (25%) study (Donovan et al. 2015) and three of the four (75%) studies described the implications of their findings (Baydala et al. 2014; Donovan et al. 2015; Lee et al. 2008).

## Discussion

This study systematically reviewed the literature on substance use prevention programs for Indigenous youth in the USA, Canada, Australia and New Zealand. The review identified 26 eligible studies, and results indicated that substance use prevention programs for Indigenous youth can reduce substance use frequency and intention to use, improve substance-related knowledge, attitudes and resistance strategies, and delay substance use initiation. In terms of program type, only one included study delivered an unadapted program directly to Indigenous adolescents, whereas all other programs were either cultural adaptations of mainstream programs, or cultural-based programs. Most studies were delivered in a school setting, either as the sole setting, or combined with family or community elements. All but five studies were delivered to a completely Indigenous participant group.

The most commonly included components in beneficial prevention programs were the inclusion of substance use education, cultural knowledge enhancement, skill development and the involvement of the community in the development of the program. The findings of this literature review should be interpreted in the light of the methodological quality of the studies, which was weak for 54% of the included studies, moderate for 27% and strong for 19% included studies. This review therefore emphasises the need for better quality evaluation studies to build a stronger evidence base around effective substance use and related harms prevention for Indigenous adolescents.

### Outcomes, Type, Setting and Context of Prevention for Indigenous Youth

Only 8% of included studies measured - intention to, and initiation of, substance use, which are important measures of successful prevention considering that every year of delaying substance use reduces the likelihood of a substance use disorder by 9% (Grant et al. 2001; Newton et al. 2014). Given the younger age of substance use initiation amongst Indigenous adolescents (Australian Institute of Health and Welfare 2006), delay of initiation should be targeted by future substance prevention programs and measured in evaluation studies.

It is promising to see that all, except one program were either culturally adapted or cultural-based programs. Cross-cultural translation of prevention concepts is important for programs to be appropriate and effective for the target group (Castro and Yasui 2017; Dickerson et al. 2018). The most common setting for programs was schools, which have the potential to reach many young people and has been identified as a priority setting for prevention (Barry et al. 2013). This setting adds complexity due to the multicultural nature of classrooms in the USA, Canada and Australia. In the USA, for example, only 13% of approximately 378,000 Native American adolescents go to an American Indian school (Bureau of Indian Education 2017), leaving most Native American students attending schools with students of other cultural backgrounds (Hecht et al. 2003). While another study identified that multicultural substance use prevention was equally effective as cultural-based prevention for students with Hispanic, European and African-American backgrounds (Hecht et al. 2003), the findings of this review suggest this may not be the case for Indigenous students. Dixon et al. (2007) argued that reservation/mission living, and a history of colonisation and dispossession have created a unique cultural context for Indigenous students that requires an appropriately tailored prevention approach. Given that most Indigenous students in the USA, Canada, Australia and New Zealand attend schools with students from a range of cultural backgrounds, the challenge for future school-based substance use prevention research is to develop programs that are culturally inclusive and effective for Indigenous students as well as students from other cultural backgrounds.

Despite the importance of family and community in Indigenous cultures (Kirmayer et al. 2003) and as a protective factor against substance use and related harms (Johnston and Thomas 2008), only one third of prevention programs identified in this review were community- or family-based. The included “family-based” programs only engaged the families through one-off workshops or pamphlets and none of the included studies evaluated the impact of the family component specifically. While family-based programs have recently been developed for Indigenous adolescents, such as the Strengthening Families Program (Kumpfer et al. 2010) and the Family Listening/Circle Program (Belone et al. 2017), none of these had published evaluations at the time of this literature review. The important role of family and community in Indigenous cultures and the evidence for the effectiveness of family- and community-based programs in non-Indigenous populations (Calabria et al. 2012; Templeton et al. 2010), highlights an area for further improvements to prevent substance use among Indigenous adolescents.

### Common Components of Effective Substance Use Prevention

The fourteen programs with beneficial substance-related outcomes for Indigenous adolescents used a combination of skill development, cultural knowledge enhancement and/or substance education. These key elements are in line with effective principles of substance use prevention for non-Indigenous populations, and it is promising that this is reflected for Indigenous adolescents (Lee et al. 2016; Newton et al. 2014). The finding that cultural knowledge enhancement was common in all effective programs highlights the importance of cultural adaptation and sensitivity to local cultural characteristics (Newton et al. 2014).

The majority of the 14 beneficial programs were developed with the local Indigenous community, a finding which aligns with international guidelines (United Nations 2008) and previous research demonstrating the importance of Indigenous ownership for effective program development (Lee et al. 2013; Snijder et al. 2015). It has now been generally accepted that Indigenous people need to be involved in every stage of the planning, implementation and evaluation of drug prevention programs (Dickerson et al. 2018).

While this review set out to assess the effectiveness of computerised and online prevention programs compared to traditional programs (Snijder et al. 2018), only two computerised programs were identified (Bowen et al. 2012; Schinke et al. 1994). This lack of use of technology in delivering substance use prevention for Indigenous adolescents is in line with findings from previous reviews and highlights an area for future development (Doran et al. 2017; Lee et al. 2013). Benefits of computerised interventions have been documented elsewhere and are thought to be especially applicable to disadvantaged populations as their flexibility can overcome issues relating to intervention implementation in hard-to-reach and culturally diverse populations (Chou et al. 2013). Given the potential for computerised programs to address issues with delivery, the high rates of technology and internet use amongst Indigenous adolescents (Garakani 2014; McNair Ingenuity Research 2014; Rice et al. 2016) and effectiveness of computer- and Internet-delivered substance use prevention in non-Indigenous populations (Champion et al. 2016), future research should explore the effectiveness of the use of computers and online technology in the delivery of substance use prevention with Indigenous adolescents.

### Methodological Considerations

This review identified five RCTs and three CCTs; however, only two of the RCTs were methodologically strong according to the critical appraisal using the EPHPP tool. This poor methodological quality likely reflects the challenges related to Indigenous-specific research as well as a lack of financial commitment in this field. Better quality research and reporting is required to improve the evidence around substance use prevention for Indigenous adolescents. Others have argued that the use of traditionally preferred research designs, such as RCTs, may be less appropriate for use with Indigenous populations (Clifford et al. 2011; Dickerson et al. 2018). Quality of evaluation research in this field can be improved by increasing the use of practical and alternative research designs, such as cluster RCTs and multiple baseline designs (Clifford and Shakeshaft 2017; Dickerson et al. 2018). Researchers and funding organisations should strive to prioritise rigorously conducted evaluation research in this field and be open to alternative designs.

Further compromising the findings of studies in this review is the lack of validated and reliable measurement tools used in evaluations, with only half of the studies using such measures. This is less than optimal considering that using measures that have not been specifically developed for use with Indigenous populations will likely under estimate the real levels of substance use (Chikritzhs and Brady 2006). The low level of use of reliable and valid measures reflects the lack of available measures developed specifically for Indigenous populations; a 2017 bibliometric review only identified 19 studies developing and/or validating drug and alcohol measurement for Indigenous populations across the USA, Australia, Canada and New Zealand between 1993 and 2014 (Clifford and Shakeshaft 2017). More research funding and efforts should be allocated to developing reliable and valid substance outcome measures for use with Indigenous populations. Such research should be developed with input from Indigenous communities about how impact can be measured, and cultural elements can be incorporated (Belone et al. 2012; Lee et al. 2018; Mushquash and Bova 2007).

### Limitations

A potential limitation of this systematic review is the Western interpretation of what constitutes beneficial outcomes. It is important to acknowledge that Western models used in this and other reviews are not the only way of knowing and that Indigenous populations in the USA (Dickerson et al. 2018), Australia (Cochran et al. 2008), New Zealand (Smith 2012) and Canada (Schnarch 2004) have their own ways of gathering and sharing knowledge that should be acknowledged and integrated into research. A barrier to integrating Indigenous ways of knowing in the current literature review was the embedded Western models in the studies included in this review. This review recommends the integration of Indigenous ways of knowing and Western research models in future substance use prevention research with Indigenous populations to ensure that the outcomes are in line with the cultural context and reflect what the local communities identify as important outcomes.

Another potential limitation is the restriction to four countries, even though, globally, there are 70 countries with Indigenous populations (United Nations 2006). It is possible that effective substance use prevention programs for Indigenous adolescents in other countries were overlooked that could be beneficial for Indigenous adolescents in the USA, Canada, Australia and New Zealand. Despite this possibility, the generalisability to the countries included in this review cannot be assumed, given the differences in their cultural and colonisation history. The review was deliberately limited to these four countries as they have a comparable history of being colonised by English settlers and are a minority in an English-dominant culture, with comparable consequences in terms of health and wellbeing outcomes (Cornell 2006). This comparability of Indigenous peoples in the USA, Canada, Australia and New Zealand makes the findings of included studies more generalisable to these four countries.

A methodological limitation of this review is the classification of studies as beneficial based on the percentage of beneficial outcomes reported in the study. This may have penalised studies for measuring more outcomes. In this review, there was a slight difference in the number of outcomes measured in studies marked as null compared to beneficial studies. On average, studies with null effects (50% or less beneficial outcomes) measured four outcomes, whereas beneficial studies (more than 50% of beneficial outcomes) measured three outcomes. While this is not ideal, it was a useful method of narratively summarising outcomes of the included studies. However, as a consequence of this approach, conclusions about effectiveness of drug prevention for Indigenous youth could not be made. Finally, this review included some studies with a small sample size and these findings should be interpreted with caution as they may lack statistical power to show a statistically significant or clinically meaningful result.

## Conclusion

Overall, the results of this review indicate that substance use prevention programs have the potential to produce beneficial substance-related outcomes for Indigenous adolescents, especially when they are developed with Indigenous people and include components of skill development, cultural knowledge enhancement and substance-related education. However, methodological quality of the included evaluations lacked the rigour required to draw conclusive statements about the effectiveness of substance use prevention programs for Indigenous adolescents. There is an urgent need for more financial and time investment in conducting rigorous evaluations using practical and alternative research designs, such as multiple baseline designs and cluster RCTs, to create a strong evidence base of what works to prevent substance use among Indigenous youth.

## Appendix

**Table 3:** Program descriptions and evaluation outcomes for included studies (*n* = 26)

First author (year)	Substances	Sample	Program	Program contributors	Setting and context	Program type	Program outcomes	
*USA*								
Allen et al. (2017)	Alcohol	100% Alaska Native 12–17 years **W1** Baseline 1, *n* = 137 **W2** Baseline 2, *n* = 121 **W3** 6 months post-W1, *n* **=** 103 **W4** 12 months post-W1**,***n* **=** 109	*Qungasvik* **INT 1** 26-modules (1–3 h), intensive delivery **INT 2** Dynamic wait-listed design, less intensive delivery Cultural enhancement, AOD protective factors	**Development** Community, research team **Facilitation** Non-prescriptive manual provided for non-trained community facilitators	Community Reservation	Culture-based	**W1vsW2** **Other**	**Null** *Peer attitudes on substance use:* INT 1 = INT 2, *ns* *Knowledge of alcohol consequences:* INT 1 = INT 2, *ns* Beneficial outcomes for suicide protection, multicultural mastery
Asdigian et al. (2016)	Cannabis	100% American Indian 12–14 years **W1** Baseline, *n =* 499 **W2** 6 months post-W1, *n* = 541 **W3** 9 months post-W1, *n* = 557 **W4** 18 months post-W1, *n* = 477	*Circle of Life* **INT** 30-h program administered over 6 months Cannabis and sexual health education, cultural enhancement, skill acquisition **CO** Waitlisted schools who received the program in phase 2 (12 months post-W1)	**Development** Community, American Indians, parents, education specialists **Facilitation** Indigenous community members, monitored by a project supervisor	School Reservation	Culture-based	**W1** **W1 > W4** **W1 < W4**	**Null** *Cannabis use:* INT < CO (*p* < 0.05) *Cannabis initiation:* INT < CO (*p* < 0.01); boys<girls (*p* < 0.05) *Cannabis use:* INT (*p* < 0.01); CO, *ns*
Bowen et al. (2012)	Tobacco	84% American Indian 12–18 years **W1** Baseline, *n* = 113 **W2** 1 month post-W1, *n* = 102	*SmokingZine American Indians* (SmokingZine) **INT** 6-week online program at a Native summer camp Tobacco education **CO** Did not receive any program	**Development** Community, youth from a diverse background **Facilitation** Online-based program	Community Reservation	Adapted	**W1 > W2** **Other**	**Beneficial** *Tobacco intended use*: INT < CO (*p* < 0.05) *Positive tobacco beliefs:* INT < CO (*p* < 0.05)Beneficial outcome for helping others quit smoking
Carter et al. (2007)^	Substance general	76% American Indian 11–12 years **W1** Baseline, *n* = 397 **W2** 6-months post-W1, *n* = 346 **W3** 18 months post-W1, *n* = 260	*Project Venture* **INT** 20-lessons, 1 h each Cultural enhancement, skill acquisition, recreational outdoor activities **CO** Did not receive any program	**Development** Not specified **Facilitation** Indigenous leaders guided by implementation manual	School Urban	Culture-based	**W1 > W3**	**Beneficial** *Substance use:* INT < CO (*p* < 0.05) *Alcohol use:* INT < CO (*p* < 0.05)
Cheadle et al. (1995)	Tobacco, cannabis, alcohol, stimulants, inhalants	91% American Indian 12–18 years **W1** Baseline, *n* = approx. 4000 **W2** 24 months post-W1, *n* = not specified **W3** 48 months post-W1, *n* = not specified	**INT** Intermittent five-year multi-component reservation program Cultural enhancement, AOD education, skill acquisition for youth **Parents** AOD education **CO** Rural control did not receive any program	**Development** Representative from the health department, schools, private non-profit organisations, Government **Facilitation** Not specified	School, community, family Reservation	Culture-based	**W1 > W3** **W1vsW3**	**Null** *Tobacco use (chewing):* INT < CO (*p* < 0.05) *Alcohol use:* INT = CO, *ns* *Tobacco use (smoking):* INT = CO, *ns* *Cannabis use:* INT = CO, *ns* *Inhalant use:* INT = CO, *ns* *Stimulant use:* INT = CO, *ns* *Alcohol initiation:* INT = CO, *ns*
Dixon et al. (2007)	Tobacco, cannabis, alcohol	16% American Indian (*separately analysed)* 11–13 years **W1** Baseline, *n* = 4222 **W2** 6 months post-W1, *n* = not specified **W3** 12 months post-W1, *n* = not specified **W4** 18 months post-W1, *n* = not specified	*keepin’ it REAL* **INT** 10-lessons, booster session 12-months post-program AOD education, skill acquisition **CO** Did not receive any program	**Development** Not specified **Facilitation** Untrained teacher	School Urban	Unadapted	**W1 < W4** **W1vsW4**	**Iatrogenic** *Alcohol use*: INT > CO (*p* < 0.05) *Cannabis use:* INT > CO (*p* < 0.05) *Tobacco use*: INT = CO, *ns*
Donovan et al. (2015)	Tobacco, cannabis, alcohol, substance use general	100% American Indian and Alaska Native 15–18 years **W1** Baseline, school *n* = 8, workshop *n* = 23 **W2** 12 months post-W1, school *n* = 6, workshop *n = 19* **W3** 16 months post-W1, school *n* = 7, workshop *n* = 19	*Healing of the Canoe* **INT school** 11-school sessions **INT workshop**s 3-off-reservation sessions Both targeted cognitive-behavioural skills, cultural enhancement **CO** No control described	**Development** Community, American Indians and Alaska Natives **Facilitation** Two trained local tribe members	School, community Reservation	Culture-based	**W1** **W1 > W2** **W1vsW3** **W1** **W1 > W2** **W1vsW2** **W1 > W3** **W1vsW3** **Other**	**Null** **School** *Alcohol use:* 63% *Tobacco use:* 50% *Cannabis use:* 50% *Substance use* (*p* < 0.05) *Substance use, ns* **Workshop** *Alcohol use:* 68% *Tobacco use:* 55% *Cannabis use:* 67% *Substance use* (*p* < 0.05) *Substance knowledge, ns* *Substance use* (*p* < 0.05) *Substance knowledge*, *ns* Beneficial outcome for self-esteem
Komro et al. (2017)	Alcohol	46% American Indian (*separately analysed*) 14–18 years **W1** Baseline, school *n =* 118, community *n* = 141, control *n* = 433 **W12** 36 months post-W1, school *n* = 220, community *n* = 198, control *n* = 571, 615 completed all surveys*****	*CONNECT* **INT school** 12 one-on-one health consultations over 3 years. Alcohol education, healthy alternatives, normative education, personal goal development *Communities Mobilizing for Change on Alcohol (CMCA)* **INT community** 6-stage program Alcohol education **INT school + community** participants were exposed to both interventions **CO** received program after the completion of this study	**Development** Not specified **Facilitation** Trained social workers, community action teams, monitored by trained staff	School, community, family Indian Territory (racially diverse)	Adapted	**W1 > W12** **Other**	**Beneficial** **School** *Alcohol use:* INT < CO (*p* < 0.01) *Binge drinking:* INT < CO (*p* < 0.05) *Alcohol consequences:* INT < CO (*p* < 0.01) **Community** *Alcohol use:* INT < CO (*p* < 0.01) *Binge drinking:* INT < CO (*p* < 0.01) *Alcohol consequences:* INT < CO (*p* < 0.01) **School + Community** *Alcohol use:* INT < CO (*p* < 0.05) *Binge drinking:* INT < CO (*p* < 0.05) *Alcohol consequences:* INT < CO (*p* < 0.05) Beneficial outcome for community prevention initiatives
Kulis et al. (2013)	Substance use general	100% American Indian 12–13 years **W1** Baseline, *n* = 57 **W2** 7 months post-W1, *n* = 57	*Living in 2 Worlds* (keepin’ it REAL) **INT** 12-lessons AOD education, skill acquisition, cultural enhancement and drug resistance strategy development **CO** No control described	**Development** Elders, students, parents, community leaders **Facilitation** Trained American Indian program leaders	School, family Urban	Adapted	**W1 < W2** **W1vsW2**	**Beneficial** *Refusal skill* (*p* < 0.05) *Explain skill* (*p* < 0.05) *Leave skill* (*p* < 0.05) *Redirect skill* (*p* < 0.05) *Avoid skill, ns* *Humour skill, ns*
Kulis et al. (2016)	Tobacco, alcohol, inhalants, cannabis.	100% American Indian 12–13 years **W1** Baseline, *n* = 107 **W2** 6 months post-W1, *n* = 91	*Living in 2 Worlds* (keepin’ it REAL) **INT** 12-lessons AOD education, skill acquisition, cultural enhancement and drug resistance strategy development **CO** Administered unadapted *keepin’ it REAL*	**Development** Elders, students, parents, community leaders **Facilitation** Trained American Indian program leaders	School, family Urban	Adapted	**W1 < W2** **W1vsW2** **Other**	**Null** *Alcohol use:* CO (*p* < 0.05) *Tobacco use:* CO (*p* < 0.05) *Substance knowledge:* INT > CO (*p* < 0.05) *Redirect skill:* (*p* < 0.05) *Alcohol use:* INT, *ns* *Tobacco use:* INT, *ns* *Cannabis use:* INT, *ns*; CO, *ns* *Inhalant use:* INT, *ns*; CO, *ns* *Substance use intent:* INT, *ns*; CO, *ns* *Alcohol amount:* INT, *ns*; CO, *ns* *Tobacco amount:* INT, *ns*; CO, *ns* *Cannabis amount:* INT, *ns*; CO, *ns* *Leave skill:* INT, *ns;* CO, *ns* *Refusal skill:* INT, *ns*; CO, *ns* *Explain skill:* INT, *ns*; CO, *ns* Beneficial outcomes for spirituality, cultural traditions, INT > CO
Lowe et al. (2012)	Substance use general	100% American Indian 13–18 years **W1** Baseline, *n* = 187 **W2** 10 weeks post-W1, *n* = 179 **W3** 23 weeks post-W1, *n* = 179	*Cherokee Talking Circle* **INT** 10-lessons Program content not described **CO** “Be A Winner” standard drug prevention curriculum	**Development** Community, Elders **Facilitation** Counsellor, cultural expert guided by intervention manual	School Indian Territory	Culture-based	**W1 > W2** **W1 > W3** **Other**	**Beneficial** *Substance-related attitudes:* INT < CO (*p* < 0.001) *Substance-related attitudes:* INT < CO (*p* < 0.001) Beneficial outcomes for total symptom severity scale, general life problem index, internal behaviour scale, external behaviour scale, self-reliance scores
Moran (1999)	Alcohol	100% American Indian 10–11 years **W1** Baseline, *n* = 71 **W2** 14 weeks post-W1, *n* = 57	*The Seventh Generation* **INT** 14-week program Cultural enhancement, AOD and depression education, skill acquisition **CO** Did not receive any program	**Development** Community, local American Indian **Facilitation** Trained American Indian staff	Community, family Urban	Culture-based	**W1** **W1vsW2**	**Null** *Alcohol use:* 20% *Positive alcohol beliefs:* INT = CO, *ns*
Moran et al. (2007)	Drug, alcohol	100% American Indian 9–13 years **W1** Baseline, *n* = 378 **W2** 13 weeks post-W1, *n* = 292 **W3** 12 months post-W1, *n* = 168	*The Seventh Generation* **INT** 13-wk program, booster sessions 6-mths post-program Cultural enhancement, AOD and depression education, skill acquisition **CO** Did not receive any program	**Development** American Indian community **Facilitation** Not specified	School, family Urban	Culture-based	**W2 > W3** **Other**	**Benifical** *Positive alcohol beliefs:* INT (*p* < 0.05) Beneficial outcomes for locus of control measure, depression scores, self-esteem and social support
Patchell et al. (2015)	Substance use general	100% American Indian 16–18 years **W1** Baseline, *n* = 44 **W2** 8.5 weeks post-W1, *n* = not specified	*Native Talking Circle Intervention* (Cherokee Talking Circle Intervention) **INT** ~ 15-lessons AOD education, healthy relationships **CO** No control described	**Development** Not specified **Facilitation** Non-local American Indian female, local tribe affiliated American Indian male	School Rural	Adapted	**W1 > W2** **Other**	**Beneficial** *Substance use* (*p* < 0.05) Beneficial outcome for self-reliance
Petoskey et al. (1998)	Tobacco, cannabis, alcohol	74% American Indian 10–18 years School: **W1** Baseline, *n* = 750 **W2** 12 months post-W1, *n* = 750 **W3** 24 months post-W1, *n* = 620 **W4** 36 months post-W1, *n* = 562 Community (not assessed) *n* = 200	*Red Cliff Wellness School & Community Curriculum, Red Cliff Community Training Curriculum* **INT school** 20–30 school lessons **INT community** 4-modules Cultural enhancement, skill acquisition **CO** waitlisted schools who received the program after the completion of this study	**Development** Not specified **Facilitation** Trained teachers, monitored by the leadership core group	School, community, family Urban	Culture-based	**W1** **W1 < W4**	**Beneficial** *Alcohol use:* 29% *Tobacco use:* 42% *Cannabis use:* 22% *Alcohol use:* INT < CO (*p* < 0.01) *Tobacco use:* INT, CO (no statistics) *Cannabis use:* INT < CO (*p* < 0.01)
Schinke et al. (1994)	Tobacco	100% American Indian 10–14 years **W1** Baseline, *n* = 368 **W2** Immediately post-lesson, *n* = 368	*Boy and Woman Bear* **INT** 1-lesson (35-min) Tobacco and diet education, cultural enhancement, skill acquisition **CO** 1-lesson (35-min) Video on general problem-solving	**Development** Not specified **Facilitation** Computer-based program	School Urban (primarily American Indian)	Culture-based	**W1vsW2**	**Null** *Tobacco knowledge:* INT = CO, *ns*
Schinke et al. (2000)	Tobacco, cannabis, alcohol	100% American Indian 9–11 years **W1** Baseline, *n* = 1396 **W2** 6 months post-W1, *n* = 1374 **W3** 18 months post-W1, *n* = 1329 **W4** 30 months post-W1, *n* = 1268 **W5** 42 months post-W1, *n* = 1199	*No program name* (Botvin LifeSkills Training) **INT** 15-lessons, booster sessions semi-annually for 3.5 years Cultural enhancement, bi-cultural competence, cognitive-behavioural skill acquisition **INT + Community** program as well as parent, teachers and wider community attended information sessions, media releases **CO** Did not receive any program	**Development** Not specified **Facilitation** Group leaders and slightly older peers	School, community, family Reservation	Adapted	**W1 > W4** **W1 > W5**	**Beneficial** *Alcohol use:* INT < CO (*p* < 0.01) *Tobacco use:* INT < INT + Community (*p* < 0.05), INT < CO (*p* < 0.05) *Alcohol use:* INT < CO (*p* < 0.01) *Tobacco use:* INT < INT + Community (*p* < 0.01), INT < CO (*p* < 0.01) *Cannabis use:* INT < CO (*p* < 0.01)
Usera (2017)	Alcohol, tobacco, substance use general	86% American Indian 10–11 years **W1** Baseline, *n* = 1531 **W2** ~ 10 months post-W1, *n* = 1145	*Lakota Circles of Hope* **INT** 10-lessons (45-min) per grade, for 4 yrs. Cultural enhancement, AOD education, skill acquisition **CO** Did not receive any program	**Development** Lakota educators **Facilitation** Indigenous or non-Indigenous trained instructor	School, family Reservation	Culture-based	**W1vsW2** **Other**	**Null** *Substance use:* INT = CO, *ns* Beneficial outcome for communication skills, cultural identity
*Australia*								
Gray et al. (1998)	Analgesic, tobacco, substance use general	100% Aboriginal and Torres Strait Islander 10–20 years **W1** Baseline, *n* = 27 **W2** 12 months post-W1, *n* = 15 **W3** 24 months post-W1, *n* = 29	*Karalundi Peer Support and Skills Training* (Elizabeth Campbell program) **INT** 10-lessons AOD and tobacco education, peer support, skill acquisition, self-esteem enhancement **CO** No control described	**Development** Community, local Aboriginal and Torres Strait Islanders **Facilitation** Not specified	School Rural	Adapted	**W1 > W3** **W1 < W3** **Other**	**Beneficial** *Analgesic use* *Substance knowledge* (qualitative) Beneficial outcomes for self-esteem, decision-making skills, health issues No statistics provided
Howard et al. (2012)^	Cannabis	100% Aboriginal and Torres Strait Islander 12–15 years **W1** Baseline, *n* = 7 **W2** 3 days post-W1, *n* = 7 **W3** 5 months post-W1, *n* = 7	*Mudyi Yindyamarra* **INT** 3-day camp Cannabis education, cultural enhancement, recreational activities **CO** No control described	**Development** Community, local Aboriginal and Torres Strait Islanders **Facilitation** Aboriginal community members, research staff	Community, family Urban	Culture-based	**W1vsW2** **W1vsW3**	***Benficial*** *Cannabis knowledge:* +34% *Cannabis knowledge:* +34%
Johnston et al. (1998)	Tobacco	95% Aboriginal and Torres Strait Islander 5–17 years **W1** Baseline, *n* = 223 **W2** Approx. 9 months post-W1, *n* = 141	*Be Smoke Free* **INT** 2-week intensive program School: Tobacco education modules Community: Recreational activities **CO** Did not receive any program	**Development** Community **Facilitation** Trained staff	School, community, family Reservation	Culture-based	**W1** **W1 < W2** **W1vsW2**	**Null** *Tobacco use: 50%* *Tobacco initiation:* 100% *Tobacco use among family*: 98% *Tobacco knowledge:* INT = CO *Tobacco use*: INT = CO No statistics provided
Lee et al. (2008)	Cannabis	100% Aboriginal and Torres Strait Islander youth **W1** Baseline, *n* = not specified **W2** 36 months post-W1, *n* = not specified	*Youth Development Unit* **INT** Program duration not specified Recreational activities, occupational training, mental health promotion, cultural enhancement **CO** No control described	**Development** Indigenous and non-Indigenous representatives, key agencies in community, local mining company **Facilitation** Case worker, Indigenous youth worker; overseen by a program co-ordinator	Community Reservation	Culture-based	**W1 > W2**	**Beneficial** *Cannabis use* (*p* < 0.01)
Malseed et al. (2014)	Tobacco, cannabis, alcohol, inhalants	90% Aboriginal and Torres Strait Islander 11–18 years **W1** Baseline, *n* = not specified **W2** 7 weeks post-W1, *n* = 103	*Deadly Choices* **INT** 7-lessons Health (nutrition, physical activity, AOD) education, skill acquisition **CO** Did not receive any program	**Development** Not specified **Facilitation** Indigenous trained leaders	School Urban	Culture-based	**W1 < W2** **W1vsW2** **Other**	**Null** *Tobacco knowledge:* INT > CO (*p <* 0.01) *Alcohol use*: INT, *ns* *Tobacco use*: INT, *ns* *Cannabis use:* INT, *ns* *Petrol use*: INT, *ns* Beneficial outcomes for chronic disease knowledge, nutrition knowledge, confidence in preventing chronic diseases, comfortable having health checks
Sheehan et al. (1995)	Alcohol	100% Aboriginal and Torres Strait Islander 12–16 years **W1** Baseline, *n* = 56 **W2** 3 months post-W1, *n* = 66	*When You Think About It Education Package* (When You Think About It) **INT** Six-lessons for grades 8, 9 and 10 Alcohol education modules **CO** No control described	**Development** Community, local Aboriginal and Torres Strait Islanders **Facilitation** Trained teachers who had attended a two-day workshop	School, family Reservation	Adapted	**W1vsW2**	**Beneficial** *Alcohol use:* 0% *Alcohol & body knowledge:* −9% *Alcohol use among friends:* +5% *Alcohol content knowledge:* +14% *Alcohol & sport knowledge: +*9% No statistics provided
*Canada*								
Baydala et al. (2014)	Alcohol	100% First Nations 11–13 years **W1** Baseline, *n* = 93 **W2** 12 months post-W1, *n* = 68	*Nimi Icinohabi* (Botvin LifeSkills Training) **INT** 3-year program, total of 23 modules, 10 booster sessions in grade 7 and 9 in grade 8 Cultural enhancement, skill acquisition, AOD education **CO** Children in the grade above acted as control and did not receive any program	**Development** Community, Sioux Nation involvement **Facilitation** Trained community program provider	School Reservation	Adapted	**W1 > W2** **W1 < W2** **Other**	**Beneficial** *Alcohol use:* INT < CO *Alcohol intended use:* INT < CO *Alcohol knowledge:* INT > CO Alcohol use positively correlated with low school attendance, beneficial outcome for self-esteem No statistics provided
Mushquash et al. (2007)	Cannabis, alcohol	100% First Nations 14–18 years **W1** Baseline, *n =* 41 **W2** Time point not specified, *n* = 25	*Nemi’simk, Seeing Oneself* **INT** 2-sessions Cognitive behavioural strategies, cultural enhancement **CO** Did not receive any program	**Development** Elders, community **Facilitation** Trained facilitators (guidance counsellors, police officers)	School Reservation	Adapted	**W1 > W2** **W1 < W2** **Other**	**Beneficial** *Alcohol use:* INT < CO (*p* < 0.05) *Cannabis use:* INT < CO (*p* < 0.05) *Alcohol abstinence:* INT > CO (*p* < 0.01) Beneficial outcome for AOD-related problems

Entries highlighted in bold relate to different waves, intervention and control groups. It is also used to highlight whether the program was beneficial and how the program was implemented. They are there to guide the reader to important information

*W1–W12* wave 1–wave 12, *INT* intervention group, *CO* control group, *AOD* alcohol and other drugs, *ns* non-significant result

Other = additional outcomes tested that are not substance use, intent to use, substance initiation, skill or knowledge-based. An equal sign indicates the INT and CO were not significantly different. ‘Drug’ is used for illicit drugs in general when programs did not specify the drugs targeted. For culturally adapted programs, the original program name is in brackets when available

*Waves 2–11 were not included in this summary table. Please refer to original paper by Komro et al. (2017) to access this data

^Grey literature paper

**Table 4:** Critical appraisal of quantitative components of included studies (*n* = 26)

First author (year)	Selection bias	Study design	Confounds	Data collection methods	Withdrawal and drop-outs	Intervention integrity	Analysis	Summary rating
USA								
Allen et al. (2017)	Moderate	Moderate	Strong	Strong	Strong	No measurement of program consistency; attrition rates were measured; no mention of other interventions influencing outcomes.	Community-level allocation and analysis; statistical methods (mixed effects regression models) were appropriate.	Strong
Asdigian et al. (2016)	Strong	Strong	Weak	Weak	Weak	Consistency of the program was measured (log books and weekly meetings); not all participants attended every session; no mention of other interventions influencing outcomes.	Organisation-level allocation and analysis; statistical methods (discrete-time survival analysis) were appropriate for risk of marijuana initiation at different ages.	Weak
Bowen et al. (2012)^[10]^	Weak	Strong	Strong	Weak	Strong	High consistency of program facilitation as it is computer-based; participant program attendance was extremely low; outcomes may be influenced by other factors occurring at the camp.	Individual-level allocation and analysis; statistical methods (*t* tests, chi-square tests) were appropriate.	Weak
Carter et al. (2007)	Weak	Strong	Weak	Weak	Moderate	Facilitators followed a program manual; attrition rates were measured; no mention of other interventions influencing outcomes.	Organisation-level allocation and analysis; statistical methods (general linear model) were appropriate.	Weak
Cheadle et al. (1995)	Weak	Strong	Strong	Strong	Weak	No description of exposure to program or consistency in delivery; outcomes likely to be influenced by other interventions taking place at the same time in the community.	Community-level allocation and analysis; statistical methods (frequencies, percentages, logistic regressions) were appropriate.	Weak
Dixon et al. (2007)	Moderate	Strong	Strong	Weak	Moderate	No description of exposure to program or consistency in delivery; attrition rates were measured; no mention of other interventions influencing outcomes.	Organisation-level allocation and analysis; statistical methods (growth curve modelling) were appropriate.	Moderate
Donovan et al. (2015)	Weak	Moderate	Strong	Strong	Strong	No description of exposure to program or consistency in delivery; attrition rates were measured; no mention of other interventions influencing outcomes.	Organisation-level allocation and analysis; statistical methods (Friedman’s two-way analysis of variance by ranks, Wilcoxon signed rank tests) were appropriate.	Moderate
Komro et al. (2017)	Moderate	Strong	Strong	Strong	Strong	Facilitators followed a program manual; attrition rates were measured; measured implementation of unaffiliated alcohol prevention efforts in community.	Community-level allocation and analysis; statistical methods (linear probability models) were appropriate.	Strong
Kulis et al. (2013)	Strong	Moderate	Weak	Weak	Strong	Consistency of program implementation was not measured; participant program attendance was not measured; no mention of other interventions influencing outcomes.	Organisation-level allocation and analysis; statistical methods (frequencies, *t* tests) were appropriate.	Weak
Kulis et al. (2016)	Moderate	Strong	Strong	Strong	Weak	Research teams attended several lessons to measure quality of instruction and fidelity to the curriculum manuals; participant program attendance was not measured; no mention of other interventions influencing outcomes.	Organisation-level allocation and analysis; statistical methods (*t* tests, Cohen’s *d*, general linear models) were appropriate.	Moderate
Lowe et al. (2012)	Moderate	Strong	Strong	Strong	Strong	Facilitators followed a program manual; attrition rates were measured; no mention of other interventions influencing outcomes.	Organisation-level allocation and analysis; statistical methods (*t* tests, general linear model) were appropriate.	Strong
Moran (1999)	Weak	Moderate	Strong	Strong	Strong	No description of exposure to program or consistency in delivery; outcomes may have been influenced by other factors in the community or school setting (i.e. school curriculum)	Community-level allocation and analysis; statistical methods (*t* tests) were appropriate.	Moderate
Moran et al. (2007)	Weak	Moderate	Strong	Strong	Weak	No description of program consistency; exposure to program described; no mention of other interventions influencing outcomes.	Community-level allocation and analysis; statistical methods (frequencies, ANOVA) were appropriate.	Weak
Patchell et al. (2015)	Moderate	Moderate	Strong	Strong	Strong	No measurement of program consistency; participant program attendance was not measured; no mention of other interventions influencing outcomes.	Community-level allocation and analysis; statistical methods (frequencies, *t* tests) were appropriate.	Strong
Petoskey et al. (1998)	Moderate	Moderate	Strong	Strong	Weak	Consistency of the program was measured; unable to measure participant attendance due to anonymous reporting; outcomes may have been influenced by other factors resulting from varying implementation sites.	Organisation-level allocation and analysis; statistical methods (ANOVA, correlations) were appropriate.	Moderate
Schinke et al. (1994)	Moderate	Moderate	Weak	Weak	Weak	High consistency of program facilitation as it is computer-based; unlikely that other factors influence outcomes as it is a one-session program.	Individual-level allocation and analysis; statistical methods (descriptives, *t* tests) were appropriate.	Weak
Schinke et al. (2000)	Moderate	Moderate	Strong	Weak	Strong	No description of consistency in delivery; attrition rates were measured; a community intervention was running simultaneously, likely to influence outcomes.	Organisation-level allocation and analysis; statistical methods (ANOVA) were appropriate.	Moderate
Usera (2017)	Moderate	Moderate	Strong	Moderate	Moderate	Consistency of the program was measured (log books, observation logs); attrition rates were measured; no mention of other interventions influencing outcomes.	Community-level allocation and analysis; statistical methods (MANOVA, ANOVA) were appropriate.	Strong
Australia								
Gray et al. (1998)	Weak	Moderate	Weak	Weak	Weak	No description of participant attendance; program consistency is unlikely as new program strategies were employed across the 2 years; no mention of other interventions influencing outcomes.	Organisation-level allocation and analysis; comparability of results was compromised by four factors: different survey questions, different points on the response scales, different levels of supervision and data was not systematically collected; no statistical analyses were conducted.	Weak
Howard et al. (2012)	Weak	Moderate	Strong	Weak	Weak	No measurement of program consistency; participant program attendance was not measured; no mention of other interventions influencing outcomes.	Community-level allocation; no statistical analysis (outcomes as percentages only).	Weak
Johnston et al. (1998)	Moderate	Moderate	Strong	Weak	Weak	Consistency of the program was not measured; not all participants attended every session; outcomes of the multi-component school and community program may have been influenced by other factors (i.e. tobacco education as part of the school curriculum).	Organisation-level allocation and analysis; minority of participants completed both surveys making statistical calculations inappropriate for comparisons.	Weak
Lee et al. (2008)	Weak	Moderate	Weak	Weak	Moderate	Many youth involved in the interventions, no information on consistency, outcomes likely to be influenced by other interventions taking place at the same time in the community (including stricter supply controls and rewards linked to school attendance).	Community-level allocation and analysis; statistical methods described in other publication. Dates of data collection (2001–2004) do not line up with dates of intervention (2003–2005), no post-test data.	Weak
Malseed et al. (2014)	Moderate	Moderate	Weak	Weak	Weak	No program consistency in delivery; no measure of participant sample size; no mention of other interventions influencing outcomes.	Organisation-level allocation and analysis; statistical methods (linear and logistic mixed-effects regression) were appropriate.	Weak
Sheehan et al. (1995)	Weak	Moderate	Weak	Weak	Moderate	No description of consistency in delivery; high absentee rates for each lesson; outcomes likely to be influenced by other interventions taking place at the same time in the community.	Organisation-level allocation and analysis; one-quarter of participants were included in analysis due to irregular attendance rate; no statistical analysis (outcomes as percentages only).	Weak
Canada								
Baydala et al. (2014)^[11]^	Moderate	Moderate	Strong	Weak	Moderate	Consistency of the program was measured; not all participants attended every session; no mention of other interventions influencing outcomes.	Organisation-level allocation and analysis; statistical methods (ANOVA, correlations) were appropriate; thematic analysis of qualitative data was appropriate.	Moderate
Mushquash et al. (2007) ^[12]^	Weak	Moderate	Weak	Strong	Moderate	Consistency of the program was measured; no mention of other interventions influencing outcomes.	Organisation-level allocation and analysis; statistical methods not described.	Weak

**Table 5:** Search strategy MEDLINE

1	((substance OR drug OR alcohol OR tobacco OR petrol OR cannabis OR kava OR methamphetamine OR MDMA OR inhalant OR marijuana OR amphetamine OR “psycho stimulant” OR smok* OR “illicit drug” OR “volatile drug”) AND (evaluat* OR effect* OR efficacy OR review OR trial) AND ((Indigenous OR Aborigin* OR “Torres Strait*” OR Maor* OR “First Nation” OR Inuit OR “American Indian*” OR “Alaskan Indian*”) AND (Austral* OR “New Zealand*” OR Canad* OR Americ*)) AND (youth OR young OR adolescen* OR teen*)).mp. AND (educat* OR prevent* OR interven* OR program).m_titl.
2	limit 1 to yr = “1990–2017”

[mp = title, abstract, original title, name of substance word, subject heading word, keyword heading word, protocol supplementary concept word, rare disease supplementary concept word, unique identifier]
